# Segmented Thermoelectric Generator under Variable Pulsed Heat Input Power

**DOI:** 10.3390/e21100929

**Published:** 2019-09-24

**Authors:** Pablo Eduardo Ruiz-Ortega, Miguel Angel Olivares-Robles, Olao Yair Enciso-Montes de Oca

**Affiliations:** 1 Instituto Politecnico Nacional, Depto. Ingenieria Bioquimica, ENCB, Ciudad de Mexico 07738, Mexico; eduardo29491@gmail.com; 2 Instituto Politecnico Nacional, SEPI ESIME Culhuacan, Ciudad de Mexico, Coyoacan 04430, Mexico; olaoyairenciso1991@gmail.com

**Keywords:** segmented thermoelectric generator, pulsed heat, transient

## Abstract

In this paper, we consider the transient state behavior of a segmented thermoelectric generator (STEG) exposed to a variable heat input power on the hot side while the transfer of heat on the cold side is by natural convection. Numerical analysis is used to calculate the power generation of the system. A one-dimensional STEG model, which includes Joule heating, the Peltier effect with constant properties of materials, is considered and governing equations are solved using the finite differences method. The transient analysis of this model is typical for energy harvesting applications. A novel design methodology, formulated on the ratio of the figure of merit of the thermoelectric materials, is developed including segmentation on the legs of the thermoelectric generator, which does not consider previous studies. In our approach, the figure of merit is an advantageous parameter to analyze its impact on thermal and electrical efficiency. The transient state of the thermoelectric generator is analyzed, considering two and three heat input sources. We obtain the temperature profiles, voltage generation, and efficiency of the STEG under pulsed heat input power. The results showed that the temperature drop along the semiconductor elements was more considerable when three pulses were applied, and when the thermal conductivity in the first segment was higher than that of the second segment. Furthermore, we show that the generated voltage and the maximum efficiency in the system occur when the value of the figure of merit in the first segment, which is in contact with the temperature source, is lower than the figure of merit for the second thermoelectric segment of the leg. The model investigated in this paper offers an essential guide on the thermal and electrical performance behavior of the system under transient conditions, which are present in many variable thermal phenomena such as solar radiation and the normalized driving cycles of an automotive thermoelectric generator.

## 1. Introduction

Power generation based on thermoelectric effects, which use new thermoelectric materials technology, is of interest for researchers because it converts thermal energy into electricity and is utilized as a new way to harvest clean energy. To satisfy world energy demands, it is vital to investigate new areas in energy conversion technologies for power generation. Recent research into the improvement of efficiency and reduced costs of thermoelectric materials make essential contributions towards harvesting clean energy [1,2]. Therefore, advances in the development of new applications to take advantage of renewable energy sources have increased in recent years and are being carried out as a new solution to reduce the use of fossil fuels [3]. Thermoelectric energy harvesting employs thermoelectric generators. A thermoelectric generator (TEG) is composed of several pairs of p-type and n-type semiconductor elements, known as legs, connected electrically in a series and thermally in parallel.

Thermoelectric generators (TEGs) generate electricity directly from thermal energy in a closed-circuit when there exists a temperature difference between the hot side and the cold side of the device, which has no moving parts. The governing thermoelectric effects in the energy conversion are the Seebeck, Peltier, Thomson, and Joule effects [4,5,6]. TEGs provides several advantages against other power generating systems such as gas-free emissions, endless shelf life, no noise, a simple structure, maintenance-free operation, no pollution [7,8,9], and have been used as a harvester of waste heat from power plants. Therefore, most of the new research focuses on improving the efficiency of TEG to reach high energy conversion [10]. Some groups of researchers have made an effort to investigate new methods for energy harvesting with several numerical models to optimize the performances of TEGs have been proposed [11,12]. Thereby, the development of promising industry and daily life applications of TEGs is expected. In the improvement of TEGs energy conversion efficiency, it is necessary to use new techniques that can be focused through device design or developing new semiconductor materials according to its thermoelectric figure of merit [13]. Geometry optimization for the legs of a thermoelectric generator has been previously analyzed by Ma et al. [14], who propose a generator with minimized thickness for maximum power output. Meng [15] showed the effects of thermocouple physical size on the performance of a thermoelectric heat pump driven by a TEG. The segmentation of the semiconductor elements improves the performance of TEGs as demonstrated by the results shown in previous research [16,17]. A segmented thermoelectric generator (STEG) contains legs composed of two thermoelectric materials and is used to take advantage of the working conditions at different temperature gradients. The efficiency of a STEG increases if the two joined thermoelectric materials are compatible, which may lead to higher efficiency. Ming et al. [18] conducted a thermal analysis, using a three-dimensional finite element model on a segmented thermoelectric generator and results indicated that maximum efficiency increased 11.2% when the load resistance value was very nearly to the internal resistance value. Shu et al. [19] propose a thermoelectric generator for engine waste heat recovery, using a three-dimensional numerical model and results showed that the output power was higher than that of a non-segmented TEG by 13.4%.

It is well known that the study of TEGs operating with pulsed heat input is more challenging in a numerical simulation than thermoelectric coolers (TECs) because both the temperature gradient along the semiconductors and the electric currents vary through time. The dynamic behavior of thermoelectric devices has been studied and their importance has been reported in works such as Paul E. Gray’s book [20]. The application of pulsed heat input is a novel method to enhance the maximum performance of thermoelectric devices and the understanding of its transient behavior is essential for optimizing energy harvesting from waste heat. Crane [21] studied the differences between TEG behavior at steady-state and transient models in a MATLAB/Simulink environment where the devices and systems modeled were optimized according to an advanced multiparameter optimization technique. Mahmoudinezhad et al. [22] studied the performance of a STEG with self-adhesive graphite sheet attached to the hot surface, under variable solar radiation at high operation temperatures using a numerical simulation by the finite volume method. Results showed that the graphite absorber had an effect on the power generation by the enhancement of absorbed radiation. Samson et al. [23] investigated a segmented asymmetrical thermoelectric generator (SASTEG) under transient conditions and the results showed that by using an asymmetrical leg the thermal stress reduced by 39.21% compared to the symmetrical leg geometry.

In transport vehicles, several studies using TEGs have been performed for waste heat recovery. For example, in driving the exhaust temperature and gas flow rate vary depending on the engine operating conditions. High temperatures are achieved in the exhaust line recovery and a portion of this wasted energy can be converted into electricity, which is advantageous. Transient tests have demonstrated that the overall power generation of a TEG can be improved by controlling the hot-side temperature [24]. In addition to this, the placement of TEGs taking into account the interaction with the internal combustion engine has also been investigated [25]. This kind of research can be carried out by using a numerical simulation. Research has also been done on the variation of solar radiation, power generation, and efficiency of a TEG under the transient condition where it has been shown the impact of the thermal contact resistance on the temperature profile and system efficiency [26]. It is very important to study the transient behavior of a TEG when the heat source is variable and even more so when using segmented materials, which is known to increase the efficiency of devices. Thermoelectric devices and thermal collectors have been studied for energy harvesting, steady-state systems, and different types of practical applications [27,28].

The previously presented literature shows the optimization of thermoelectric generators using segmented legs. Measurements of figure of merit has been investigated using different techniques and by also considering different leg materials (inhomogeneous or of an irregular shape), as well as configurations of TEGs, observing an increase in the figure of merit due to the bulk thermoelectric effect [29,30,31]. Previous studies do not take into account the relationship of the figure of merit between two different materials using variable pulsed heat input to optimize TEG performance. This paper focuses on the application of the segmented thermoelectric materials of a TEG when exposed to a variable pulsed heat input for different energy harvesting applications.

## 2. One-Dimensional Model of a Segmented Thermoelectric Generator (STEG)

**Figure 1:**
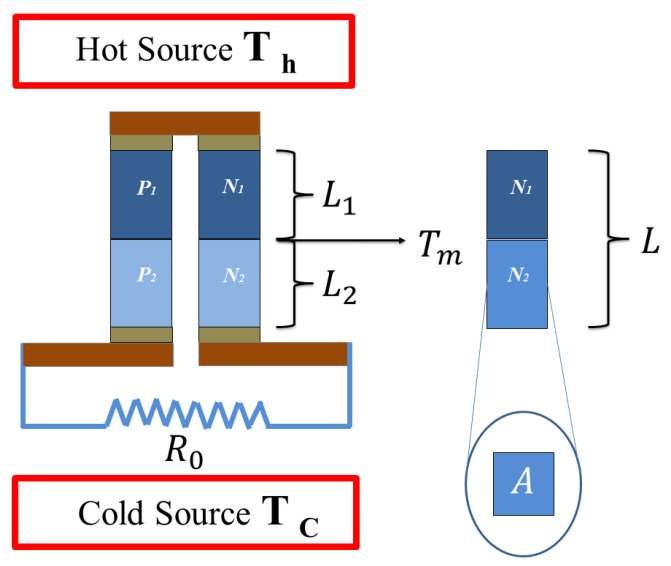
1D model of a segmented thermoelectric generator (STEG) under variable heat input power. $L_{1}$ and $L_{2}$ are the length for the first and second segment of the leg, respectively. $P_{1}$, $N_{1}$ and $P_{2}$$N_{2}$ are the p-type and n-type elements for the first and second segment, respectively, and $R_{0}$ is the load resistance.

In this work, numerical analysis based on the finite differences method is developed to study a STEG’s transient characteristics for example, spatial temperature profiles, voltage output, and efficiency. The proposed model for the segmented legs of the STEG is shown in Figure 1, where *A* is the cross-sectional area, $L_{1}$ and $L_{2}$ are the lengths of the first and second segment, respectively, $L=L_{1}+L_{2}$ is the total length, $T_{c}$ and $T_{h}$ are the temperatures at the cold and heat ends of the thermoelectric element, and $T_{m}$ is the temperature at the junction of the two segments. Numerical solutions for the spatial temperature profiles, power output, and efficiency are carried out considering variable heat input pulses when the STEG is exposed to (a) two heat input sources, and (b) three heat input sources.

## 3. Numerical Model

The thermoelectric phenomena are described using (a) the energy balance equation,

(1) $$DC_{p}\frac{\partial T}{\partial t}+\nabla \cdot q^{\prime \prime }=Q^{\prime }$$

where $C_{p}$ is the specific heat capacity, *D* is the density, *t* is the time, and *T* is the temperature.

$Q^{\prime }$ is the Joule heating energy,

(2) $$Q^{\prime }=JV$$

$q^{\prime \prime }$ is the input heat flux,

(3) $$q^{\prime \prime }=-\kappa \nabla T+\Pi J$$

Π is the Peltier coefficient, *V* is the difference in electric potential, and *J* is the electric current flux.

(b) is the current density continuity,

(4) $$\frac{\partial \rho_{c}}{\partial t}=\nabla \cdot J$$

and $\rho_{c}$ is the charge density.

It is well known that,

(5) Π=αT

and

(6) J=−σ∇E−σα∇T

where σ is the electrical conductivity, κ is the thermal conductivity, *E* is the electric field, and α is the Seebeck coefficient.

The energy balance to the thermoelement (n-type and p-type) using the 1D unsteady-state heat transfer modeling is given by:

(7) $$\frac{\partial^{2}T}{\partial x^{2}}+\frac{\rho_{1,2}I^{2}}{\kappa_{1,2}A^{2}}=\frac{D_{1,2}C_{p1,p2}}{\kappa_{1,2}}\frac{\partial T}{\partial t}$$

where ρ, *I*, and *x* are the electrical resistivity, the electric current flowing in a closed-circuit, and spatial coordinate, respectively. The subscripts determine the type of material, where 1 is for the $CoSb_{3}$ and 2 is for the $Bi_{2}Te_{3}$.

Equation (8) represents the energy balance for $CoSb_{3}$ and $Bi_{2}Te_{3}$ at the interface. Equation (9) represents the energy balance for the cold side of the thermoelement exposed to natural convection and is expressed as follows:

(8) $$\frac{\Delta xAD_{1}C_{p1}+\Delta xAD_{2}C_{p2}}{2}\frac{\partial T}{\partial t}=-\alpha_{1}TI+\kappa_{1}A\frac{\partial T}{\partial x}+\alpha_{2}TI-\kappa_{2}A\frac{\partial T}{\partial x}$$

(9) $$\frac{\Delta xAD_{2}C_{p2}}{2}\frac{\partial T}{\partial t}=-\alpha_{2}TI+\kappa_{2}A\frac{\partial T}{\partial x}+hA\left(T-T_{a}\right)$$

where $h=2.5\frac{W}{mK}$ is the natural convection coefficient and $T_{a}$ is the ambient temperature [21].

### 3.1. Initial Conditions

The initial conditions on the lower and upper surface of the thermoelement are as follows:

(10) $$T_{h}\left(t,L\right)=T_{h}\left(t,0\right)=388\;K\;T_{c}\left(t,L\right)=T_{c}\left(0,L\right)=298\;K$$

### 3.2. Transient State Equations Solution by the Finite Differences Method

The finite difference method has been used to solve the differential equation with the transient term (7) since this method is stable and more accurate than other methods. Further details about this numerical technique to solve the finite difference equations are in: Numerical Methods for engineers, Steven C. Chapra (2007). All different temperature profiles for the transient state are obtained by applying the finite differences method to the energy balance equations. The thermoelement have been divided into *N* number of equal parts with equivalent length (Δx) and node numbers from i=0 to i=N+1, where N=110.

Solving Equations (8)–(10), we obtain,

(11) $$T_{i-1}^{n+1}\left(\frac{\kappa_{1,2}\Delta t}{C_{p1,p2}D_{1,2}\Delta x^{2}}\right)-T_{i}^{n+1}\left(\frac{2\kappa_{1,2}\Delta t}{C_{p1,p2}D_{1,2}\Delta x^{2}}+1\right)+T_{i+1}^{n+1}\left(\frac{\kappa_{1,2}\Delta t}{C_{p1,p2}D_{1,2}\Delta x^{2}}\right)+T_{i}^{n}+\frac{\rho_{1,2}I^{2}}{A}=0$$

Equation (11) describes the temperature distribution for $CoSb_{3}$ and $Bi_{2}Te_{3}$.

(12) $$T_{i-1}^{n+1}\left(\frac{\kappa_{1}A\Delta t}{\psi \Delta x}\right)-T_{i}^{n+1}\left(\frac{\alpha_{2}I\Delta t}{\psi }+\frac{\kappa_{2}A\Delta t}{\psi \Delta x}+\frac{\kappa_{1}A\Delta t}{\psi \Delta x}-\frac{\alpha_{1}I\Delta t}{\psi }-1\right)-T_{i+1}^{n+1}\left(\frac{\kappa_{2}A\Delta t}{\psi \Delta x}\right)+T_{i}^{n}=0$$

Equation (12) describes the temperature $CoSb_{3}$ and $Bi_{2}Te_{3}$ at interface.

(13) $$T_{i-1}^{n+1}\left(\frac{2\kappa_{2}A\Delta t}{C_{p2}D_{2}\Delta x^{2}}\right)-T_{i}^{n+1}\left(\frac{2\kappa_{2}\Delta t}{C_{p2}D_{2}\Delta x^{2}}+\frac{2h\Delta t}{D_{2}C_{p2}\Delta x}-\frac{2\alpha_{2}I\Delta t}{D_{2}AC_{p2}\Delta x}-1\right)+T_{i}^{n}-\frac{2hT_{a}\Delta t}{D_{2}C_{p2}\Delta x}=0$$

where ψ is given as:

(14) $$\psi =\frac{\Delta xAD_{1}C_{p1}+\Delta xAD_{2}C_{p2}}{2}$$

Equation (13) describes the temperature of the surface exposed to the natural convection of the $Bi_{2}Te_{3}$ material.

### 3.3. Electrical Performance Equations

In closed-circuit mode, the generated output voltage is given by:

(15) $$V_{cc}=V_{oc}-IR$$

where $V_{oc}$ is the open-circuit voltage and can be determined with the following equation:

(16) $$V_{oc}=T_{0}\alpha_{E}-T_{L}\alpha_{E}$$

The effective coefficient of Seebeck is calculated for a segmented semiconductor material [32] and is defined in Equation (17) as follows:

(17) $$\alpha_{E}=\alpha_{1}\frac{T_{0}-T_{\frac{N}{2}+1}}{T_{h}-T_{c}}+\alpha_{2}\frac{T_{\frac{N}{2}+1}-T_{N+1}}{T_{h}-T_{c}}$$

where $T_{0}$, $T_{\frac{N}{2}+1}$ and $T_{N+1}$ are the hot side constant temperature, interface temperature through time, and cold side temperature through time, respectively.

The electrical resistance, *R*, of the thermoelement is given by:

(18) $$R=\frac{\rho_{1}L}{A}+\frac{\rho_{2}L}{A}$$

where *L* is the length of the thermoelement of the STEG. Considering a resistive load $R_{0}$ = 1.5 Ω [33], the electric current can be determined with the following equation:

(19) $$I=\frac{V_{oc}}{R_{0}+R}$$

In a thermoelectric power generation device, efficiency is given by,

(20) $$\eta =\frac{W}{Q_{h}}=\frac{I(\alpha_{E}\Delta T-IR)}{K_{th}\Delta T+\alpha_{E}IT_{h}-\frac{1}{2}I^{2}R}$$

where *W* is the power delivered by the TEG system, $Q_{h}$ is the heat flow from the heat source to the sink, and $K_{th}=\kappa A/L$ is the thermal conductance.

### 3.4. The Figure of Merit

To achieve a high value of the figure of merit, the dimensionless parameter that determines the performance of a thermoelectric material requires high electrical conductivity, low thermal conductivity, and a high Seebeck coefficient.

The figure of merit is defined as follows:

(21) $$Z_{1,2}=\frac{\alpha_{1,2}^{2}}{\rho_{1,2}\kappa_{1,2}}$$

Equation (21) is used to know the merit figure of material 1 and 2.

(22) $$Z_{r}=\frac{Z_{1}}{Z_{2}}$$

where $Z_{r}$ is the ratio of material 1’s figure of merit to material 2’s figure of merit.

## 4. Material Properties and Geometry Description

In this paper, we assume that the thermoelectric materials’ properties are independent of temperature and the constant parameters and dimensions of the semiconductor elements are given in Table 1. It has been proven in other works that by using constant material properties for calculations, thermal and electrical characterization can be matched with experimental data [33]. It has been proven that the Thomson effect does not affect the temperature profile in TEGs, but the output voltage is impacted by the Thomson effect and even more so by the load resistance. Here we focus on optimization according to the ratio of figure of merit $Z_{r}$ since the value of the figure of merit changes, in small ranges, due to the variation of the average temperature. Therefore, the temperature gradient along the semiconductor’s elements is the most dominant factor and hence thermal and electrical characterization can be achieved [34]. Optimization that takes into account the Thomson effect is presented in this study. A novel design methodology using a computational tool that focuses on examining the influence of segmentation is proposed. Depending on the particular application for energy harvesting, TEG performance varies mainly due to α, κ, τ, and ρ which in this paper are considered in $Z_{r}$ and $Z_{r,\tau }$, without the Thomson effect and with the Thomson effect, respectively. Therefore, and according to this last statement, only a resistive load $R_{0}$ is considered to show the thermal and electrical response of a STEG considering different values of the figure of merit. Thomson coefficient values are assumed as constant and Seebeck is related to the Thomson effect as follows:

(23) $$\alpha_{1,2}=\left(\alpha_{p(1,2)}-\alpha_{n(1,2)}\right)+\left(\tau_{p(1,2)}-\tau_{n(1,2)}\right)ln\left(\frac{T_{avg}}{T_{ref}}\right)$$

where $T_{ref}$ is the room temperature (298 K) and $T_{avg}$ is the average temperature when the heat fluxes are applied, i.e., 468 K.

## 5. Heat Input Power Effect on Performance

**Figure 2:**
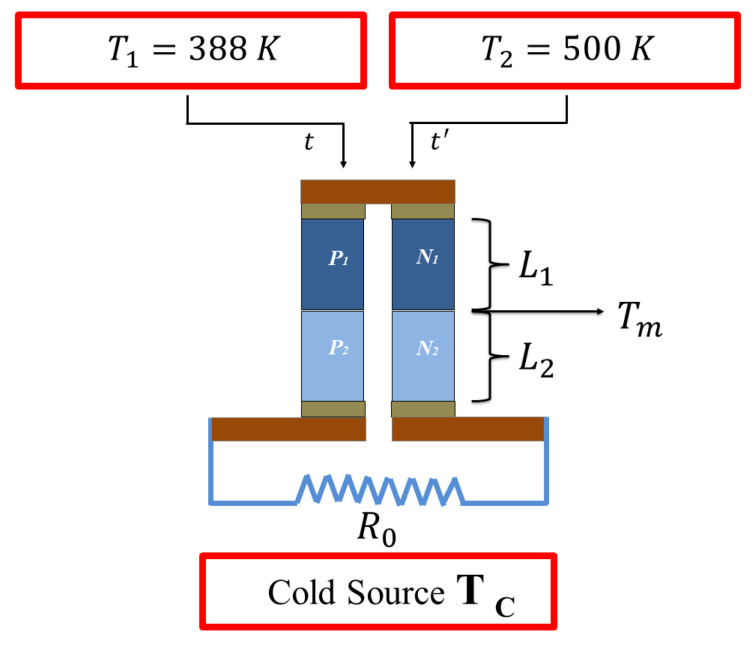
Two temperature sources are applied on the thermoelements at time *t* and $t^{\prime }$.

A thermoelectric generator consists of copper conductors that connect the p-type and n-type semiconductor materials and facilitates electrical conductivity within the TEG. In this work, these copper conductors as well as heat losses due to radiation and transverse convection are not taken into account, but an external load resistor, and thus voltage output of the STEG, can be measured. For calculations, all materials are assumed homogeneous, and property materials such as thermal conductivities, electrical resistivity, the Thomson coefficient, and materials’ specific heat capacities are assumed constant and do not change throughout time or temperature. We considered a length of the semiconductor element in the STEG of $L_{1,2}=1.1\times 10^{-3}$ m for the first and second segment. Variable heating conditions are applied to the hot side of the TEG while the cold side changes through time. We calculated the spatial temperature profiles along the STEG elements, the cold side temperature, and output voltage during pulse heat operation under the following conditions:

(a) The system begins at room temperature at 298 K;

(b) The heat pulses are input to the system, alternating the temperature sources $T_{1}=388$ K for some time of t= 0–5 s and then;

(c) A source of $T_{2}=500$ K for a period of time of $t^{\prime }=$ 5–10 s, as shown in Figure 2.

**Figure 3:**
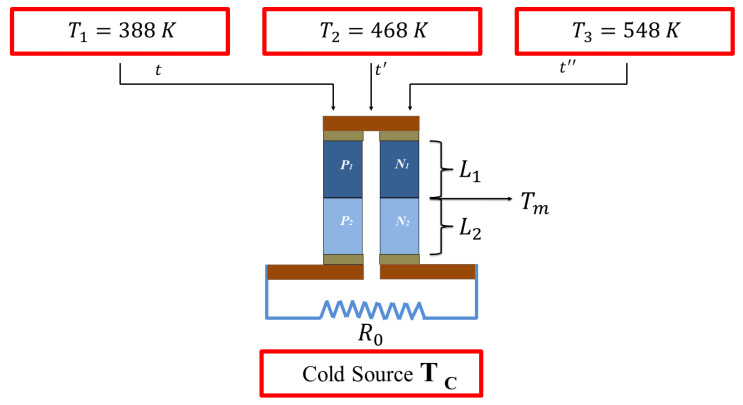
Three temperature sources are applied on the thermoelements at time *t*, $t^{\prime }$, and $t^{\prime \prime }$.

This process is repeated up to a total time of 35 s. For the second case (b), firstly a temperature source of $T_{1}=388$ K is applied, then a temperature source of $T_{2}=468$ K, and finally a temperature source of $T_{3}=548$ K, with each source applied in a period of time of *t* = 0–5 s, *t* = 5–10 s, and *t* = 10–15 s, respectively, as shown in Figure 3. This process is repeated up to a total time of 50 s. In both cases, $CoSb_{3}$ is placed in the first segment and $Bi_{2}Te_{3}$ in the second segment. The load resistance is kept constant throughout this study. Rectangular heat input flux function is used to model the pulse in all cases. In our simulation model, the relevant constant parameters used in the numerical simulation are listed in Table 1.

### 5.1. Two Heat Input Pulses: Thermal Behavior

**Figure 4:**
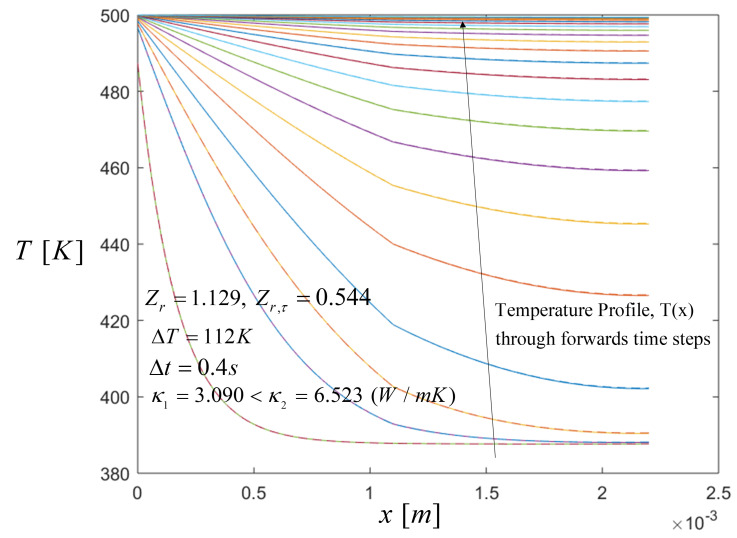
Spatial temperature profile during the heat pulse when $Z_{r}=1.129$ and $Z_{r,\tau }=0.544$, with Thomson and without Thomson coefficients, respectively. Comparison results with the Thomson effect (dashed lines) and without the Thomson effect (solid lines). The arrow direction point out the temperature distribution along the thermoelements through the time steps.

In particular, the output voltage and the temperature profiles across a STEG under periodic pulse heating were compared for different $Z_{r}$ and $Z_{r,\tau }$ values, for both case (a) and (b). In all temperature and voltage profiles, solid lines do not take into account the Thomson effect and dashed lines are for results taking into account the Thomson effect. According to Equations (11)–(13), Figure 4 shows the spatial temperature profile evaluated when the heat pulse of 500 K was applied in a period of time of *t* = 5 s, along the STEG elements until the system reached a steady-state distribution for case (a) as previously described. Here the temperature profile behavior was similar in all heat pulses for all cases, and so there was no need to include all the figures. For this result a temperature difference of ΔT=112 K when the heat input pulse of 500 K was applied is used, and the temperature distribution was evaluated until the temperature in the segment reached a steady-state distribution.

It can be seen that the temperature increases on the hot side, i.e., in the first segment, at the moment when the heat pulse was applied which caused an increase in the temperatures in a specific length and here no change in the interface was observed for the first instants of time. After 0.25 s there was a change at the interface because the value of the thermal conductivity of the $CoSb_{3}$ was higher than the material $Bi_{2}Te_{3}$, $\kappa_{1}=3.09$ W/mK and $\kappa_{2}=6.523$ W/mK respectively, and therefore heat was preferentially conducted toward the cross-sectional area easily in the first segment. Notice that the heat absorbed in the first segment was due to $CoSb_{3}$ material, and the heat released in the second segment was due to $Bi_{2}Te_{3}$.

**Figure 5:**
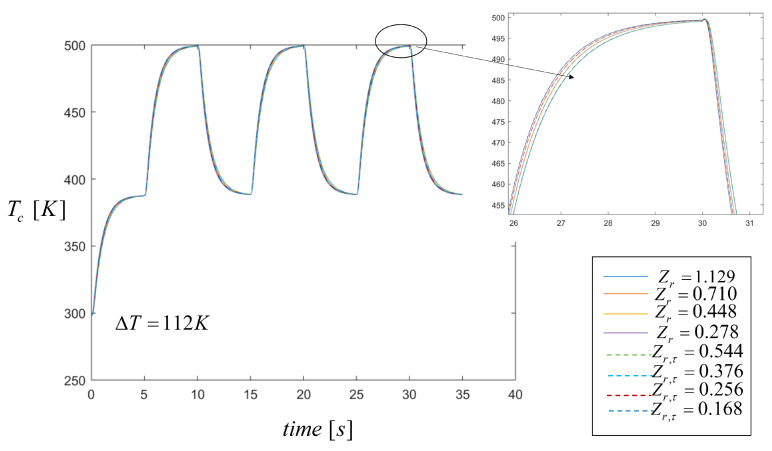
Temperature distribution on the cold side through time when two heat pulses are applied. Comparison results with the Thomson effect (dashed lines) and without the Thomson effect (solid lines).

In this paper, the thermal characterization was made considering open-circuit conditions. The application of the two transient rectangular pulsed heat power is shown in Figure 5 which shows the transient behavior of the cold side temperature, $T_{c}$, for different $Z_{r}$ and $Z_{r,\tau }$ values shown in the figure. As shown from these results, the temperature profiles were not affected by the Thomson effect as is well known from the literature and only output voltage results change considering the Thomson effect, as demonstrated in the next section. To show the periodic behavior of the system when the pulses were repeatedly applied, the total time in all simulation was set to 35 s. Simulations were performed by varying the thermal conductivity of the $Bi_{2}Te_{3}$ material to obtain the different values of $Z_{r}$ and this range spanned the cases when $\kappa_{1}<\kappa_{2}$ to $\kappa_{1}>\kappa_{2}$. It can be seen from these results that the cold side temperature $T_{c}$ values were very similar for all $Z_{r}$ values where the thermal conductivity, electrical resistivity, and Seebeck coefficient of the $CoSb_{3}$ material remained as constant values, i.e., $Z_{1}=cte$, and only material properties of $Bi_{2}Te_{3}$ were changed in order to vary $Z_{2}$ values.

### 5.2. Electrical Responses to Periodic Heat Fluxes

**Figure 6:**
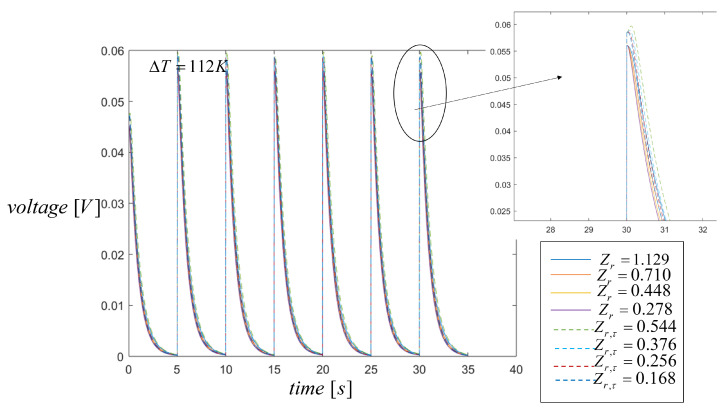
Voltage generation when two heat input pulses are applied with a duty cycle of 35 s. Comparison results with the Thomson effect (dashed lines) and without the Thomson effect (solid lines).

According to the law of heat transfer from the hot side to the cold side, the temperature of the cold side changes until thermal equilibrium is attained within a STEG. Temperature gradient values become smaller through time when the system approaches steady state and therefore when the generated voltage also decreases. Maintaining $T_{c}$ values constant in order to achieve higher temperature gradients is difficult which directly affects the electrical performance of the TEG. Higher voltage generation accuracy can be achieved by considering the heat input power as an efficient parameter to analyze its influence on the energy conversion of a STEG. As is well known from the literature, no previous study has focused on the effect of variable pulsed heat on the performance of a STEG as is done in this work. The duty cycle determines the ratio of the heating time to the period time in the simulation if the duty cycle value is very high, the TEG performance is reduced. In this case for the two heat pulses, the period of heating is 5 s and the period time is 10 s, then the duty cycle is set to 0.5 s, which is the value used in all the simulations. The heating effects on the maximum voltage generation are shown in Figure 6 for different $Z_{r}$ and $Z_{r,\tau }$ values. The output voltage follows Ohm’s law, i.e., it is given by the current multiplied by the load resistance from Equation (19). It can be seen that during one period of pulse heating the voltage generation values, for all the different $Z_{r}$ and $Z_{r\tau }$, are very similar in all cases reaching a maximum value of $V_{max}=55$ mV and $V_{max}=59$ mV, without the Thomson effect and with the Thomson effect respectively. The difference in the energy generated by STEG under periodic heating is almost the same, with a difference of less than 0.5% which can be easily seen from Figure 6, indicating that working with two input pulses and changing the figure of merit of the second material, i.e., $Z_{2}$, does not significantly affect the voltage generation. These results indicate that the smaller the value of $Z_{r}$, the more output voltage in the system is generated, which is confirmed in the next section where changes in the figure of merit when working with three heat pulses, increase the voltage generated.

### 5.3. Three Heat Input Pulses: Thermal Behavior

**Figure 7:**
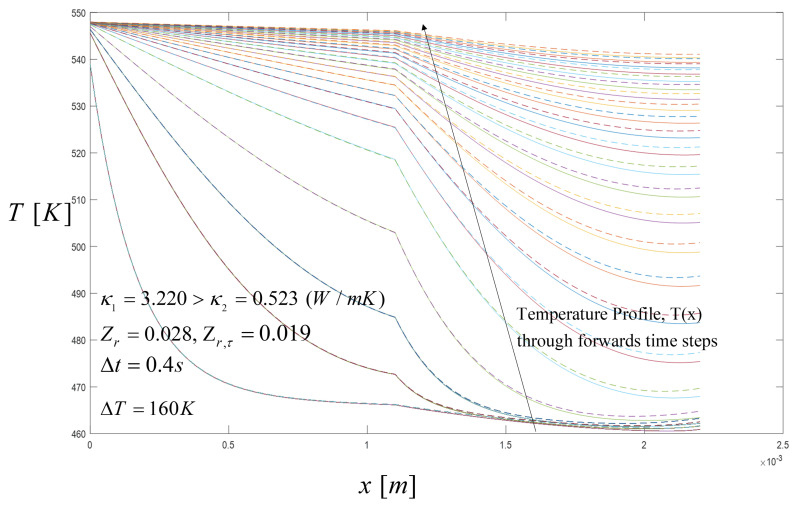
Spatial temperature profile during the pulse from 10.25 s to 15 s in time intervals of 0.25 s with $Z_{r}=0.028$ and $Z_{r,\tau }=0.019$, with Thomson and without Thomson coefficients, respectively. Comparison results with the Thomson effect (dashed lines) and without the Thomson effect (solid lines). The arrow direction point out the temperature distribution along the thermoelements through the time steps.

In this section, we analyze the case in which three heat input pulses are applied, i.e., case (b) previously described, where the initial temperature for the STEG system is 298 K. The spatial temperature profiles were affected by the heat pulses applied and the properties of the materials. Thereby, we calculated the temperature profiles through the time when $Z_{r}=0.028$ and $Z_{r,\tau }=0.019$. In the next section, we will show that this value improves the voltage output compared with other $Z_{r}$ and $Z_{r,\tau }$ values. Figure 7 shows the spatial temperature profile, evaluated when the heat pulse of 548 K is applied during 5 s until the system reaches a steady-state distribution. In this case, for three heat pulses, the time of heating is again 5 s and the period time is 15 s, then the duty cycle is set to 0.33 s. We should note that for the case when $\kappa_{1}>\kappa_{2}$, the heat conduction is better in the first segment and from the interface forward to the second segment, the conduction of heat is reduced due to the lower thermal conductivity of the semiconductor material in the second segment. The higher temperature of the third pulse significantly changes the temperature profile, which can be appreciated from the interface when the *Z* values changes i.e., $Z_{1}<Z_{2}$, compared to the case for two heat pulses.

**Figure 8:**
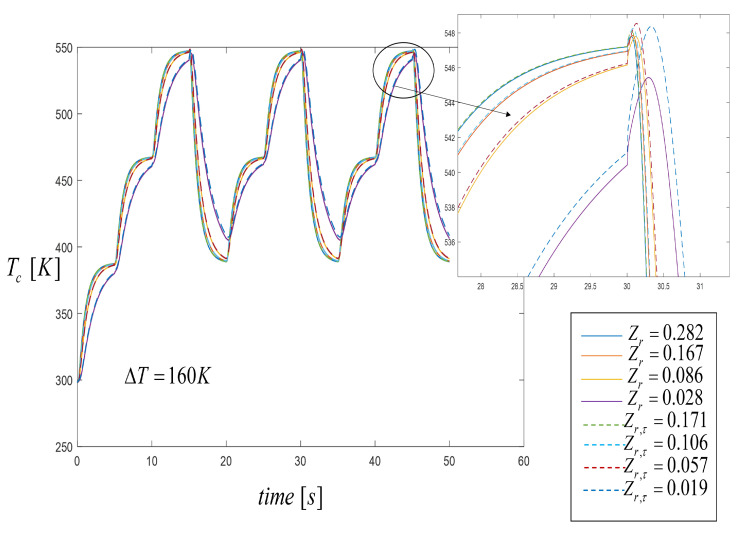
Temperature distribution on the cold side through time when three heat pulses are applied. Comparison results with the Thomson effect (dashed lines) and without the Thomson effect (solid lines).

Figure 8 shows the temperature profiles on the cold side, $T_{c}$, for different $Z_{r}$ and $Z_{r,\tau }$. For the case when $Z_{r,\tau }=0.019$, the temperature profile of the cold side showed a small increase in temperature at the moment when the source at 500 K was replaced by the source at 388 K. It is worthy to note that depending on the thermal conduction coefficient and considering the Thomson effect, which directly affects $Z_{r,\tau }$ values, the system reached its higher temperature values for $T_{c}$, due to the amount of heat flux across the interface for the case when $\kappa_{1}>\kappa_{2}$. For all the other cases, the temperature profiles were close to each other as shown in Figure 8. From these results we can point that the spatial temperature profile played an essential role in power generation. In fact, there was a relation between $Z_{r}$ and $Z_{r,\tau }$ values with the output voltage and efficiency of the STEG system for the different temperature profiles. See the next section.

### 5.4. Electrical and Efficiency Responses to Periodic Heat Fluxes

**Figure 9:**
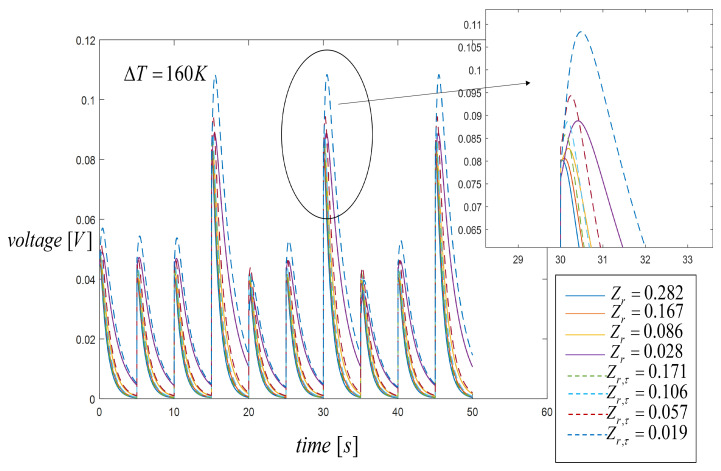
Voltage generation when three heat input pulses are applied with a duty cycle of 50 s. Comparison results with the Thomson effect (dashed lines) and without the Thomson effect (solid lines).

By using a third heat input pulse, higher values on the spatial temperature profiles were reached and consequently the output voltage increased, as shown in Figure 9 when $Z_{r}=0.0028$ and $Z_{r,\tau }=0.019$. The two important parameters in power generation are the figure of merit and temperature difference between the hot and cold sides of the STEG. Our results showed that a lower figure of merit value was required in the second segment to achieve a higher output voltage. In this paper, a small variation of the STEG output voltage was observed, since the value of the figure of merit changed within a small range in all other cases of $Z_{r}$ values. For this case, the difference in the energy generated by the STEG changed under three periodic heating compared with the case for two input pulses. Figure 9 shows that working with three input pulses and reducing the devalue of the second material’s figure of merit directly affected the voltage generation. Maximum output voltages were $V_{max}=88.7$ mV, without the Thomson effect, and $V_{max,\tau }=108.3$, with the Thomson effect. As shown in the figure, the output voltage was significantly affected for the Thomson effect, with an 18.1% increase of voltage generated by the system considering the Thomson effect. Thus, the importance of including the Thomson effect was critical to the optimum characterization of STEG models in the transient state.

**Figure 10:**
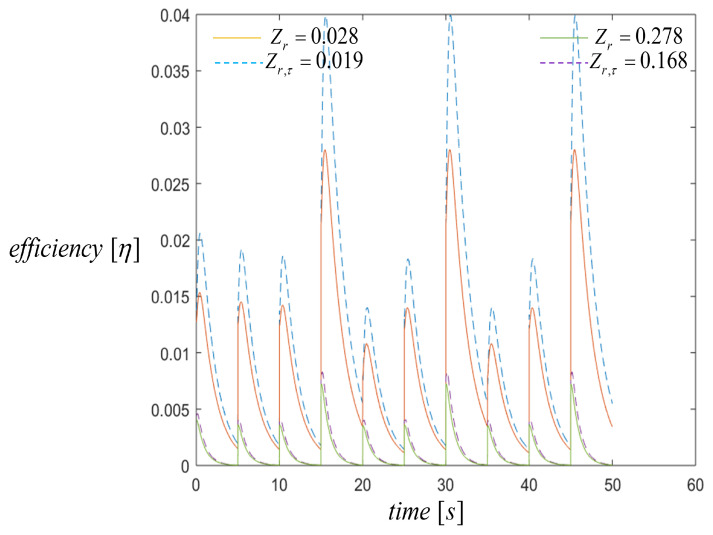
Efficiency when three heat input pulses are applied with a duty cycle of 50 s for the cases when $Z_{r}=0.287$ and $Z_{r,\tau }=0.168$, lower efficiency, and $Z_{r}=0.028$ and $Z_{r,\tau }=0.019$, maximum efficiency. Comparison results with the Thomson effect (dashed lines) and the without Thomson effect (solid lines).

From Figure 10, for transient state efficiency, we can see that maximum efficiency could be obtained when there exists a maximum temperature difference following the voltage behavior. Results show that the highest efficiency values were 2.8% and 4%, for $Z_{r}=0.028$ (orange line) and $Z_{r,\tau }=0.019$ (blue line) values, respectively. Therefore, the lower value of the ratio of the figure of merit was needed for maximum efficiency. In both cases for the two first pulses, 388 K and 468 K, the voltage was nearly half of the peak voltage for the pulse of 548 K. These results show that the new design for thermoelectric generator incorporating the segmented materials under variable heat input pulse must take into account the variation of temperature differences through time. The efficiency increased to a maximum of 4%, but then decreased to reach a steady-state when the pulses were changed to consider the Thomson effect. The efficiency was highest in agreement with the output voltage results. The numerical simulation shows the voltage and efficiency values obtained for the system in transient state. In general, the energy generated was significantly affected by the ratio of the figure of merit $Z_{r}$. In a real power generation scenario, the heat input to the device was not always controlled to hold the temperature differential. Thus, we modeled a system behavior using variable heat input.

## 6. Conclusions

In this paper, a numerical investigation of a STEG under variable pulsed heat input power was performed. The electrical performance, thermal behavior, and efficiency were obtained for different values of the ratio $Z_{r}$ and $Z_{r,\tau }$. Simulations were performed by varying the thermal conductivity and Seebeck coefficient of $Bi_{2}Te_{3}$. We gained different values of $Z_{r}$ and the results showed optimal $Z_{r}$ values for better system performance. The equations for the numerical method based on the finite-difference analysis were developed and solved in MATLAB. The most relevant conclusions and results of this study are the following.

In the segmented thermoelement, the value of the figure of merit for the first segmented, on which the temperature source was directly applied, must be lower than the figure of merit value of the second material in order to obtain better performance. In our case the materials used in the segmented thermoelement were $CoSb_{3}$ and $Bi_{2}Te_{3}$.

From spatial temperature profile, it was observed that when the figure of merit $Z_{1}<Z_{2}$, the system required more time to reach steady-state. This is because the $Bi_{2}Te_{3}$ thermoelectric properties such as conductivity and the Seebeck coefficient directly affected the figure of merit. Our results proved that when the value of the figure of merit $Z_{2}$ increased, the voltage generated also increased, thus reaching its highest value.

Cold side temperature profiles through time showed that when $Z_{r,\tau }=0.019$ the highest temperature was reached $T_{c}=548.4$ K at the moment in which the temperature source of 548 K was replaced by the source of 388 K, therefore under these conditions a maximum ΔT between the hot side and the cold side was reached.

The optimum performance was obtained considering the Thomson effect when $Z_{r,\tau }=0.019$, where the figure of merit of $CoSb_{3}$ is $Z_{1}=1.9\times 10^{-3}$ and $Bi_{2}Te_{3}$ is $Z_{2}=93\times 10^{-3}$ and the maximum difference of temperature was ΔT=160.4 K. The maximum voltage reached when considering the Thomson effect was $V_{max,\tau }=108.3$ mV which was 18.1% higher in comparison to the voltage generated without the Thomson effect, Vmax=88.7 mV.

## Figures and Tables

**Table 1:** Constant properties of the thermoelectric materials [22,33].

Property	Material 1 ${\mathrm{CoSb}}_{3}$	Material 2 ${\mathrm{Bi}}_{2}{\mathrm{Te}}_{3}$	Unit
α	$459\times 10^{-6}$	$512\times 10^{-6}$	V K${}^{-1}$
κ	3.22	3.518	W m${}^{-1}$ K${}^{-1}$
ρ	$1.01\times 10^{-5}$	$4.378\times 10^{-5}$	Ωm
*D*	7582	8160	Kg m${}^{3}$
$T_{a}$	298	298	K
*A*	$2.25\times 10^{-6}$	$2.25\times 10^{-6}$	m${}^{2}$
*L*	$2.2\times 10^{-3}$	$2.2\times 10^{-3}$	m
$C_{p}$	238.7	155	J kg${}^{-1}$ K${}^{-1}$
τ	$157\times 10^{-6}$	$22.394\times 10^{-6}$	V K${}^{-1}$
